# Structuring the AI-enabled home learning environment: a gatekeeper model of digital capital, trust, and relational support

**DOI:** 10.3389/fpsyg.2026.1838910

**Published:** 2026-06-22

**Authors:** XiaCheng Song, Huafeng Qu, Lu Sun, Jing Jin, XiQiong Yi, Junfeng Zhu, Huirong Huang

**Affiliations:** 1 School of Information and Intelligent Engineering, Yunnan College of Business Management, Kunming, China; 2 Xi‘an Fanyi University, Xi'An, China; 3 Guangdong University of Science and Technology, Dongguan, China; 4 Dongguan Polytechnic, Dongguan, China; 5 Zhaoqing University, Zhaoqing, China; 6 Guangdong Industry Polytechnic University, Guangzhou, China

**Keywords:** AI trust, AI-enabled home learning environment (AI-HLE), digital divide, home–school collaboration, parental digital literacy, parental mediation

## Abstract

**Introduction:**

As AI tools increasingly enter family life, parents function as gatekeepers who may shape whether AI becomes part of a governable learning ecology or remains an unregulated convenience. This study examined how family background, digital capital, AI-related beliefs, and relational support are associated with parental behavioral intention and willingness to pay in the AI-enabled home learning environment.

**Methods:**

We surveyed 585 Chinese parents of children from preschool through secondary school, and 567 valid responses were analyzed. An associational structural path model with observed composite variables was estimated, linking socioeconomic status, household AI use, parental digital literacy, cultural capital, AI trust, privacy concerns, algorithmic awareness, parental mediation, and home-school collaboration to behavioral intention and willingness to pay.

**Results:**

Household AI use was positively associated with parental digital literacy but did not consistently relate to broader digital capital or governance readiness. Socioeconomic status was associated with downstream support primarily through parental digital literacy, which was related to higher trust and, via relational supports, to behavioral intention. Willingness to pay was interpreted more cautiously as a financial-intention outcome. The model explained more variance in behavioral intention than in willingness to pay, and subgroup analyses indicated broadly comparable structural patterns across sample-defined lower- and higher-SES groups.

**Discussion:**

These findings suggest that equity-oriented AI-in-education initiatives should prioritize parents' digital capability, calibrated trust, and relational infrastructures that enable families to govern, not merely consume, AI in children's learning. Because the data are cross-sectional, the findings should be interpreted as associations rather than causal pathways.

## 1 Introduction

Over the past few years, AI technologies including recommendation algorithms, intelligent tutoring systems, and more recently generative AI have moved rapidly from experimental pilots into children's everyday learning environments. Policy documents, industry roadmaps, and much of the research discourse now frame AI as a key driver of personalized and data-informed education. Yet most of this attention has centered on schools, platforms, and students, implicitly treating AI adoption as something that happens “in the classroom” or “on the platform” (Mustafa et al., 2024). In practice, however, the integration of AI is largely mediated at home: acting as gatekeepers, parents determine not only whether AI is adopted, but whether it is embedded in a governable learning ecology or merely tolerated as an unregulated convenience. Despite this gatekeeping role, we still know relatively little about how parents, as everyday decision-makers, come to trust, question, or support AI-supported learning for their children.

This literature can be read as three related but still fragmented streams. Indeed, research examining the digital divide and digital capital suggests significant variance in families in terms of device access, connectivity, and digital skills, and these inequalities inform children's learning opportunities (Singh et al., 2022; Ventrella and Cotnam-Kappel, 2024). However, many existing studies treat access and frequency of use as ends in themselves, implicitly assuming that greater exposure naturally translates into the capacity to govern these tools. This overlooks the possibility that high-frequency usage may reflect convenience-driven consumption rather than the digital competence required to scaffold children's learning (Singh et al., 2022; Ventrella and Cotnam-Kappel, 2024). A second line of work relying on technology acceptance and adoption models focuses on general adoption constructs such as perceived usefulness and perceived ease of use, without clarifying whether parental support is primarily inhibited by perceived risks or associated with trust grounded in digital competence, a distinction with direct implications for intervention design (Balaskas et al., 2025; Lim et al., 2025; Shrivastava, 2025). Finally, a third category of literature has identified parental mediation and the home–school nexus as being critical to children's use of digital media, but has seldom included these relational resources in a broader AI adoption trajectory that links family background, digital capital, psychological mechanisms, and resource-allocation decisions related to the home learning environment (e.g., willingness to financially support AI-enabled learning resources) (Balleys, 2022; Li et al., 2022; Lin et al., 2021). Consequently, we do not yet have a unified understanding of how resources, beliefs, and relationships work together to influence parents' engagement with AI-supported learning.

To conceptualize the home context, we draw upon learning environment frameworks (Fraser, 1998; Moos, 1980). These frameworks posit that any learning environment comprises three fundamental dimensions: Relationship Dimensions (the nature of interpersonal support), Personal Development Dimensions (opportunities for growth), and System Maintenance and Change Dimensions (order and responsiveness). While traditionally applied to classrooms, this lens is critical for the “AI-enabled Home Learning Environment” (AI-HLE). In this study, we map parental mediation and home-school collaboration onto the Relationship Dimensions; parents' digital literacy and AI trust onto the psychosocial climate that supports personal development; and family SES and digital capital onto the system maintenance and resource foundations. This theoretical grounding clarifies why the model includes both resource-based and relational paths rather than treating AI use as a simple technology-adoption decision (Fraser, 2011).

This study addresses these gaps by specifying and estimating a multilevel framework of family AI adoption in the context of schooling from kindergarten to secondary education in China. We integrate four sets of constructs: (a) family background, captured by an SES index and an AI use index; (b) digital capital, represented by parental digital literacy and cultural capital; (c) AI-related beliefs, including AI trust, privacy concerns, and perceived algorithmic/learning risk; and (d) relational resources, namely parental mediation and home–school collaboration, linked to parental behavioral intention to support AI-enhanced learning and willingness to financially support AI-enabled learning resources. Using a structural path model with survey data from 585 parents, we examine how background factors are associated with digital and cultural resources, how these in turn relate to differentiated AI-related beliefs, and how these beliefs are linked to relational practices and downstream behavioral outcomes. The model structure is theory-led: family background is placed upstream as a resource condition, digital literacy and cultural capital are treated as capacity indicators, AI trust and risk-related beliefs represent psychosocial appraisal, and parental mediation and home-school collaboration represent relational supports. We also examine whether the core structural relations are broadly comparable across sample-defined lower- and higher-SES groups.

Against this backdrop, the present study addresses the following research questions:

**RQ1**. How are environmental resources (digital capital) and the psychosocial climate (AI trust/risk) associated with the relational dimensions (parental mediation/collaboration) of the AI-enabled home learning environment?

**RQ2**. To what extent do digital capital and AI-related beliefs statistically mediate the associations between family background and parents' behavioral intention to support AI-enabled learning in the home learning environment and their willingness to financially support AI-enabled learning resources?

**RQ3**. To what extent do parental mediation and home–school collaboration statistically mediate the links between AI-related beliefs and parents' behavioral intention to support AI-enabled learning in the home learning environment and their willingness to financially support AI-enabled learning resources?

**RQ4**. To what extent are the structural associations in the AI-enabled home learning environment broadly comparable across sample-defined SES subgroups?

## 2 Method

### 2.1 Study design

This study used a cross-sectional, questionnaire-based design to examine how family background and home learning conditions relate to parents' AI-related perceptions, parenting practices, and downstream behavioral outcomes (Bonifacci et al., 2024; Zhang et al., 2025). Due to the cross-sectional design, causal interpretations cannot be established; all estimated paths are interpreted as theory-informed statistical associations. The analytic framework was specified as a structural path model with observed composite variables, allowing the hypothesized relations among socioeconomic resources, AI exposure, home learning resources, psychosocial climate, relational support processes, and behavioral outcomes to be examined within a single coherent modeling system (Kline, 2016; Lampropoulos et al., 2023).

### 2.2 Participants and procedure

Data were collected via an online survey administered to parents (or primary caregivers) of children from preschool (pre-K) through secondary school. A total of 585 questionnaires were initially collected between November 1, 2025 and December 31, 2025. The recruitment procedure should be understood as a voluntary, non-probability online survey rather than a population-representative sampling design. To ensure response quality, the survey included a prespecified attention-check item with an explicit instruction to select a particular response option; 18 respondents failed this check and were excluded (Oppenheimer et al., 2009). After quality control, 567 responses were retained for analysis. Participation was voluntary, informed consent was obtained electronically before the survey began, and responses were recorded and analyzed anonymously (World Medical Association, 2013).

### 2.3 Measures

All constructs were measured using the study questionnaire and operationalised as observed scores for analysis. Unless otherwise noted, items were rated on a 5-point Likert-type scale (1 = strongly disagree, 5 = strongly agree), with higher scores indicating higher levels of the corresponding construct; background indicators (e.g., education and income) were recorded as ordered categories (Babaoglu et al., 2025; Likert, 1932). Parental digital literacy (PDL) was computed as the mean of three items (C2–C4; e.g., “I understand the basic functions and limitations of common AI tools,” “I can set appropriate app permissions and time-management rules for my child,” and “I can select suitable AI applications based on my child's subject needs”). Cultural capital (CC) was assessed with a single item (D1) focused on support for extracurricular reading and cultural activities. AI trust (AIT) was computed as the mean of two items (E1–E2; trusting AI to recommend appropriate learning content and trusting AI tools recommended by schools or authoritative institutions). Privacy concerns (PRC) were assessed with two items (E3–E4; concerns about excessive data collection and non-transparent downstream data use). Algorithmic awareness (AA) was assessed with three items capturing perceived AI-related learning risks and opacity cues (E5–E7; concerns about overreliance, reduced creativity/independent thinking, and non-transparent decision processes that may cause unease or misleading outcomes). Relational supports were measured as observed scores: parental mediation (PM) was assessed with a single item (F1; co-use and discussion of AI learning tools with the child), whereas home–school collaboration (HS) was computed as the mean of two items (F3–F4; desire for school-provided parent guidance/training and willingness to communicate with teachers about suitable AI tools). Behavioral intention (BI; G1) and willingness to pay (WTP; G2) were each assessed with a single item. Single-item indicators were used only for narrow, concrete focal outcomes or practices rather than for broad latent domains; this choice reduced respondent burden in an online parent survey but necessarily limits construct breadth and reliability assessment. An attention-check item (D3) was used solely for quality control and was not included in any construct scoring (Ng et al., 2024).

### 2.4 Composite construction and scoring

Composite variables were computed prior to model estimation. For SES and AI use, indices were first constructed at the respondent level (by aggregating their prespecified indicators) and then z-standardized to align with the index-based conceptualization and facilitate comparability across components (OECD et al., 2008). For the remaining constructs, observed composite scores were computed by averaging the corresponding items (or retained as single-item indicators where applicable). These scoring decisions were specified from item content and the conceptual model, not derived after inspecting structural results. For structural path modeling, all observed constructs used in the structural model were subsequently z-standardized (mean = 0, SD = 1), which is reflected in the “_z” variable naming convention in the model outputs (e.g., SES_z, PDL_z). Descriptive statistics in Table 1 are reported on the original (unstandardized) score metrics for interpretability.

**Table 1:** Descriptive statistics and reliability for study constructs (*N* = 567).

Construct	Items	Mean	SD	Reliability
SES index	2	3.09	0.60	— (Composite)
AI use index	2	3.22	0.58	— (Composite)
Digital literacy (PDL)	3	4.12	0.53	— (Composite)
Cultural capital (CC)	1	4.37	0.71	—
AI trust (AIT)	2	3.86	0.66	0.46
Privacy concerns (PRC)	2	3.39	1.20	0.86
Algo awareness (AA)	3	3.49	1.12	0.86
Parental mediation (PM)	1	4.11	0.71	—
Home-school collab (HS)	2	4.13	0.67	0.52
Behavioral intention (BI)	1	4.19	0.75	—
Willingness to pay (WTP)	1	4.17	0.79	—

SD, standard deviation. Reliability reports Cronbach's alpha for multi-item scales. “— (Index)” indicates index-type constructs for which internal-consistency reliability is not applicable. “— (Composite)” indicates an observed composite score for which internal-consistency reliability is not reported in the present table. “—” indicates single-item measures, for which internal-consistency reliability cannot be estimated.

### 2.5 Data screening and missing data handling

Data screening followed the prespecified quality-control rule based on the attention check, resulting in the exclusion of 18 invalid responses and an analytic sample of *N* = 567. Subsequent analyses were conducted using full information maximum likelihood (FIML) to handle missingness in the observed variables, allowing all available information to contribute to parameter estimation under the missing-at-random assumption without listwise deletion (Enders, 2022).

To assess potential common method bias, we conducted Harman's single-factor test using an unrotated factor solution across the primary self-report items included in the model (Podsakoff et al., 2003, 2012). The first factor accounted for 23.3% of the total variance, which is below the conventional 50% threshold, suggesting that common method bias was unlikely to be a dominant threat in this dataset (Fuller et al., 2016; Podsakoff et al., 2003).

### 2.6 Structural model specification and evaluation criteria

The hypothesized model was estimated as a structural path model using observed composite variables in the lavaan package in R (Rosseel, 2012). Robust maximum likelihood estimation (MLR) was used to obtain standard errors and test statistics that are less sensitive to non-normality. Because several constructs were represented by single-item or brief composite indicators, this analytic strategy was intended to evaluate the overall pattern of structural associations parsimoniously rather than to estimate a full latent-variable SEM with explicit separation of measurement error. Accordingly, the model should be read as an observed-variable path analysis and not as evidence that the measurement model has fully established latent construct validity. Model fit was evaluated using χ^2^, χ^2^/df, the comparative fit index (CFI), the Tucker–Lewis index (TLI), the root mean square error of approximation (RMSEA) with its 90% confidence interval, and the standardized root mean square residual (SRMR) (Jobst et al., 2022; Savalei and Rosseel, 2022). Direct effects are reported as standardized path coefficients (β) with 95% confidence intervals; statistical significance was inferred when the confidence interval did not include zero.

### 2.7 Mediation and multi-group analyses

Indirect effects were examined using non-parametric bootstrap resampling (5,000 draws) with bias-corrected and accelerated (BCa) 95% confidence intervals; an indirect effect was interpreted as supported when its confidence interval did not include zero (Pesigan and Cheung, 2023; Tofighi and Kelley, 2020). To explore whether the structural associations were broadly comparable across sample-defined SES subgroups, a multi-group structural path analysis was conducted by splitting the sample into relatively lower- and higher-SES groups using the median of the SES index (Cheung and Rensvold, 2002; Iacobucci et al., 2015). A configural model and a regression-constrained structural model were compared, and group differences were evaluated using ΔCFI and a likelihood ratio test (Cheung and Rensvold, 2002; Putnick and Bornstein, 2016). Given the sample composition and median-split grouping strategy, these subgroup analyses were treated as exploratory rather than as evidence of sharply differentiated socioeconomic strata (Cheung and Rensvold, 2002).

## 3 Results

### 3.1 Sample characteristics and AI use patterns in the home learning environment

As summarized in Table 2, the final analytic sample comprised 567 parents. Children were primarily in primary school (55.20%, *n* = 313), followed by preschool (31.92%, *n* = 181) and secondary school (12.87%, *n* = 73). Most parents reported a bachelor's degree (78.48%, *n* = 445), with smaller proportions holding a master's degree (9.70%, *n* = 55), an associate degree (9.17%, *n* = 52), a high school education or below (2.29%, *n* = 13), or a doctoral degree (0.35%, *n* = 2). Household income was most frequently in Category 3 (44.27%, *n* = 251), followed by Category 4 (23.99%, *n* = 136), Category 2 (17.99%, *n* = 102), Category 5 (10.93%, *n* = 62), and Category 1 (2.82%, *n* = 16). The majority of respondents resided in Region Category 1 (83.42%, *n* = 473), with fewer in Category 2 (14.99%, *n* = 85) and Category 3 (1.59%, *n* = 9); most children attended School Type Category 1 (89.59%, *n* = 508). Regarding AI use, children most commonly fell into Use Frequency Category 2 (38.98%, *n* = 221) or Category 3 (35.63%, *n* = 202), whereas parents most often reported AI use in Status Category 1 (64.73%, *n* = 367) or Category 2 (33.16%, *n* = 188).

**Table 2:** Participant characteristics (*N* = 567).

Variable	Category	*n*	Percent
Child educational stage	1	181	31.92%
	2	313	55.20%
	3	73	12.87%
Parent education	1	13	2.29%
	2	52	9.17%
	3	445	78.48%
	4	55	9.70%
	5	2	0.35%
Household income	1	16	2.82%
	2	102	17.99%
	3	251	44.27%
	4	136	23.99%
	5	62	10.93%
Region type	1	473	83.42%
	2	85	14.99%
	3	9	1.59%
School type	1	508	89.59%
	2	59	10.41%
Child AI use frequency	1	16	2.82%
	2	221	38.98%
	3	202	35.63%
	4	111	19.58%
	5	17	3.00%
Parent AI use status	1	367	64.73%
	2	188	33.16%
	3	11	1.94%
	4	1	0.18%

Values are reported as *n* and percentage (%). Percentages may not sum to 100% due to rounding. Category codes correspond to the questionnaire response options.

### 3.2 Descriptive statistics, reliability, and correlations among key constructs

Table 1 presents descriptive statistics and reliability for the key study constructs (*N* = 567). Mean levels were relatively high for Digital Literacy (PDL); (*M* = 4.12, SD = 0.53), Cultural Capital (CC); (*M* = 4.37, SD = 0.71), Parental Mediation (PM); (*M* = 4.11, SD = 0.71), Home–School Collaboration (HS); (*M* = 4.13, SD = 0.67), Behavioral Intention (BI); (*M* = 4.19, SD = 0.75), and Willingness to Pay (WTP); (*M* = 4.17, SD = 0.79), while AI Trust (AIT) was moderately positive (*M* = 3.86, SD = 0.66). Privacy Concerns (PRC); (*M* = 3.39, SD = 1.20) and Algorithmic Awareness (AA); (*M* = 3.49, SD = 1.12) exhibited comparatively greater dispersion. Internal consistency was strong for PRC and AA (Cronbach's α = 0.86 for both). For the brief two-item AIT (α = 0.46) and HS (α = 0.52) composites, reliability was relatively low and should be interpreted cautiously. Cronbach's alpha is sensitive to item number and can underestimate internal consistency for very short scales, especially two-item measures, but the low values still indicate that these composites should not be treated as fully validated latent scales. In the present study, these brief indicators were retained as pragmatic observed measures within an observed-composite structural path model. Likewise, several constructs (e.g., CC, PM, BI, and WTP) were operationalized with single items to capture relatively concrete focal dimensions while limiting respondent burden; prior methodological work suggests that single-item indicators can be acceptable when the target construct is narrow and unambiguous. Nevertheless, these measurement choices reduce construct breadth, preclude internal-consistency estimates for single-item variables, and should temper interpretation of the structural and mediation results.

### 3.3 Conceptual model and hypothesized pathways

**Figure 1:**
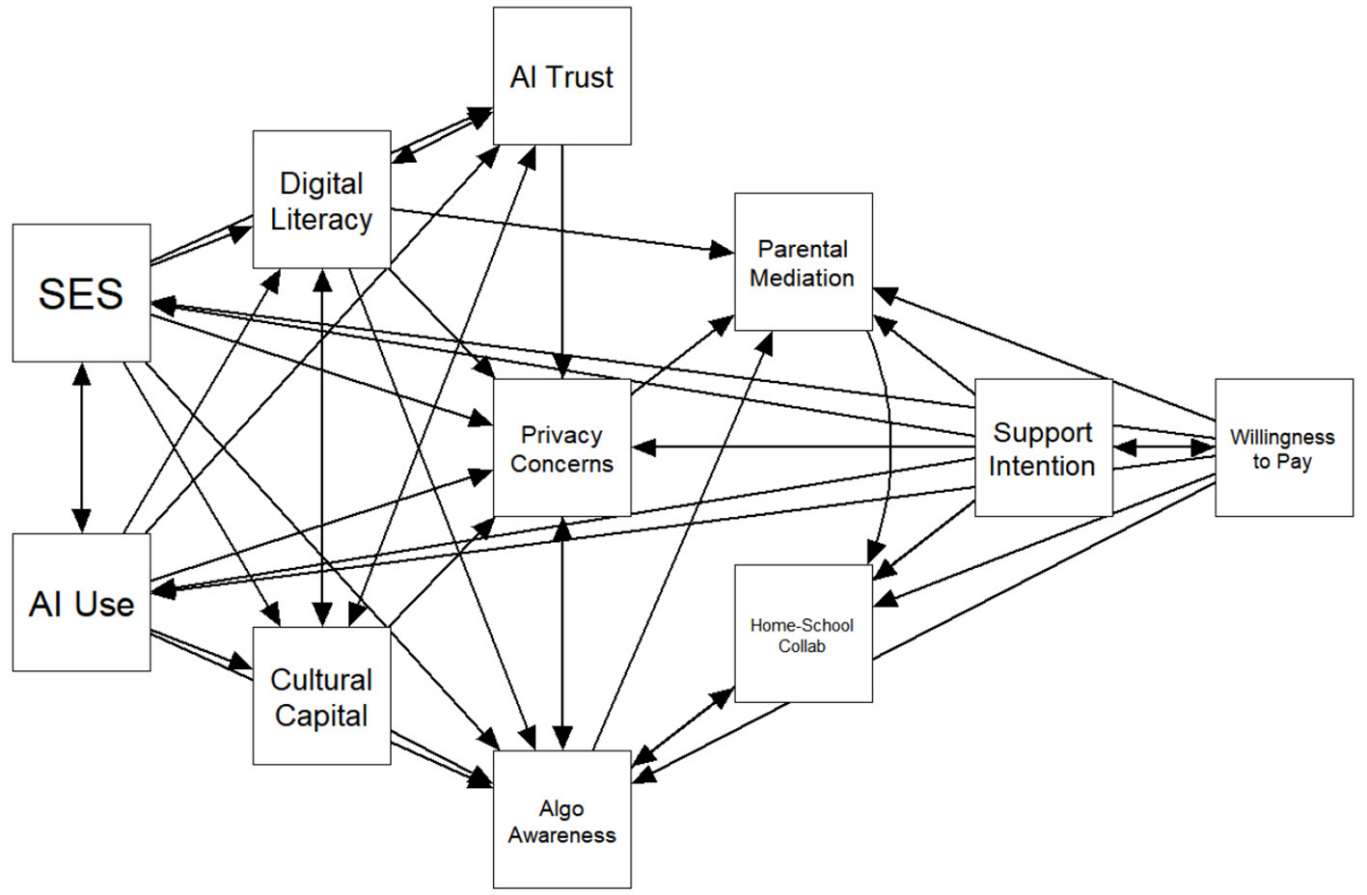
Conceptual model for the structural path analyses. SES index and AI use index represent family background factors. PDL and CC represent digital capital. AIT, PRC, and AA denote AI-related psychological factors (AI trust, privacy concerns, and algorithmic awareness). PM and HS reflect relational resources (parental mediation and home–school collaboration). BI and WTP represent parental behavioral outcomes. Bidirectional arrows represent covariances; single-headed arrows represent theory-informed directional associations in the statistical model.

Figure 1 presents the conceptual framework guiding the structural path analyses and clarifies the rationale for the main path groups. The model positions family background factors (SES index and AI use index) as conceptually upstream because socioeconomic resources and household exposure shape the conditions under which parents encounter, evaluate, and use AI tools. Parental digital literacy and cultural capital represent digital-capital resources through which background conditions may be associated with AI-related appraisals. AI trust, privacy concerns, and algorithmic awareness are placed before relational supports because parents' beliefs about AI plausibly shape how they mediate children's use and coordinate with schools. These upstream constructs are examined in relation to downstream parental outcomes (behavioral intention and willingness to pay), while recognizing potential conceptual overlap among competence, trust, and relational support. Single-headed arrows represent theory-informed directional associations in the statistical model, while bidirectional arrows indicate covariances among constructs; they should not be interpreted as evidence of causal ordering in the cross-sectional data.

Alternative structures were considered during model specification, including simpler models that omitted relational mediators or treated willingness to pay as a direct outcome. The retained specification was selected because it aligned most closely with the learning-environment framing and allowed the analysis to distinguish resource, belief, relational, and outcome components while keeping the model parsimonious.

### 3.4 Structural model fit

As reported in Table 3, the structural path model estimated with robust maximum likelihood (MLR) and FIML for missing data showed an acceptable but imperfect pattern of fit. The scaled chi-square test was significant (χ^2^_scaled = 58.50, df = 15, *p* < 0.001), with χ^2^/df = 3.90. CFI was acceptable (CFI_robust = 0.955), whereas TLI was comparatively low (TLI_robust = 0.836). RMSEA indicated reasonable approximation error [RMSEA_robust = 0.077, 90% CI (0.057, 0.098)], and SRMR suggested acceptable residual-based fit (SRMR = 0.051). Taken together, these indices support cautious interpretation of the structural associations rather than strong claims of close model fit.

**Table 3:** Model fit indices for the structural model (MLR, FIML).

Index	Value
Chi-square (Scaled)	58.50
df	15
*p*-value	0.000
Chi/df	3.90
CFI (Robust)	0.955
TLI (Robust)	0.836
RMSEA (Robust)	0.077
RMSEA 90% CI	[0.057, 0.098]
SRMR	0.051

Fit indices were computed from the structural path model estimated with robust maximum likelihood (MLR) and full information maximum likelihood (FIML) for missing data.

**Figure 2:**
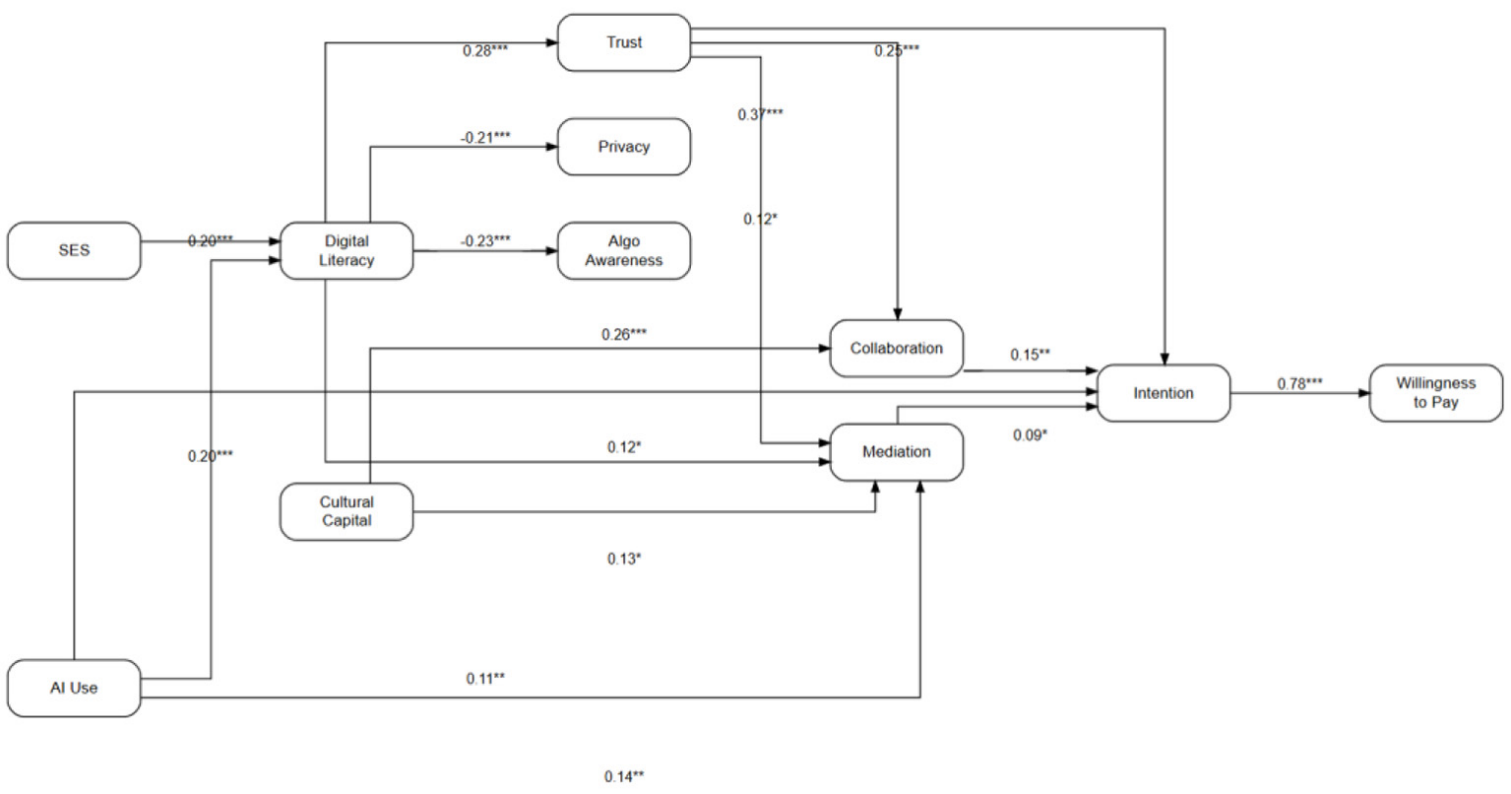
Final structural path model with standardized coefficients for statistically significant pathsValues are standardized path coefficients (β). Only statistically significant regression paths are displayed for visual clarity (*p < 0.05, **p < 0.01, ***p < 0.001). Covariances among conceptually related variables were included in the estimated model but are omitted from the figure.

Figure 2 presents the statistically significant standardized regression paths from the final structural path model. Consistent with the estimated structure, both SES (β = 0.204) and AI use (β = 0.202) are positively associated with digital literacy, while neither SES nor AI use significantly predicts cultural capital in this specification. Digital literacy is positively related to AI trust (β = 0.278) and negatively related to privacy concerns (β = −0.214) and algorithmic awareness (β = −0.230). Downstream, parental mediation was positively associated with digital literacy (β = 0.257) and AI trust (β = 0.119), and home–school collaboration was most clearly associated with AI trust (β = 0.374). In predicting outcomes, behavioral intention was positively associated with home–school collaboration (β = 0.155), AI trust (β = 0.247), and AI use (β = 0.138), and willingness to pay was positively associated with behavioral intention (β = 0.776). Given the cross-sectional design and the single-item WTP measure, this WTP association should be interpreted cautiously rather than as evidence of a causal effect or a complete explanation of financial commitment.

### 3.5 Structural paths and mediation effects

Table 4 summarizes the standardized structural paths (β, std.all) with 95% CIs. SES [β = 0.204, 95% CI (0.110, 0.298)] and AI use [β = 0.202, 95% CI (0.111, 0.294)] were both positively associated with parental digital literacy (PDL), whereas neither SES [β = 0.078, 95% CI (−0.008, 0.165)] nor AI use [β = 0.069, 95% CI (−0.027, 0.165)] significantly predicted cultural capital (CC). Higher PDL predicted greater AI trust [AIT; β = 0.278, 95% CI (0.178, 0.378)] and lower privacy concerns [PRC; β = −0.214, 95% CI (−0.296, −0.132)] and algorithmic awareness [AA; β = −0.230, 95% CI (−0.307, −0.153)], while CC showed no significant links to AIT, PRC, or AA. Downstream, parental mediation (PM) was positively predicted by PDL [β = 0.257, 95% CI (0.164, 0.350)], AIT [β = 0.119, 95% CI (0.022, 0.216)], CC [β = 0.131, 95% CI (0.024, 0.238)], and AI use [β = 0.114, 95% CI (0.031, 0.197)]. Home–school collaboration (HS) was most clearly associated with AIT within the fitted path model [β = 0.374, 95% CI (0.272, 0.476)] and CC [β = 0.116, 95% CI (0.023, 0.209)]. In predicting behavioral intention (BI), HS [β = 0.155, 95% CI (0.049, 0.260)], AIT [β = 0.247, 95% CI (0.152, 0.340)], and AI use [β = 0.138, 95% CI (0.048, 0.227)] were significant, whereas PM was not [β = 0.088, 95% CI (0.000, 0.175)]. Finally, willingness to pay (WTP) was positively associated with BI [β = 0.776, 95% CI (0.358, 1.196)], with no significant direct effects of HS, SES, or AI use on WTP; this finding is reported as an observed association and should not be overinterpreted given the narrow WTP measurement and likely omitted economic factors.

**Table 4:** Structural path estimates (standardized coefficients).

DV	IV	Beta (Std.)	Sig	95% CI
PDL_z	SES_z	0.204	^*^	(0.110, 0.298)
PDL_z	AI_Use_z	0.202	^*^	(0.111, 0.294)
CC_z	SES_z	0.078	ns	(−0.008, 0.165)
CC_z	AI_Use_z	0.069	ns	(−0.027, 0.165)
AIT_z	PDL_z	0.278	^*^	(0.178, 0.378)
AIT_z	CC_z	0.052	ns	(−0.041, 0.146)
PRC_z	PDL_z	−0.214	^*^	(−0.296, −0.132)
PRC_z	CC_z	−0.017	ns	(−0.103, 0.069)
AA_z	PDL_z	−0.230	^*^	(−0.307, −0.153)
AA_z	CC_z	−0.009	ns	(−0.093, 0.075)
PM_z	PDL_z	0.257	^*^	(0.164, 0.350)
PM_z	AIT_z	0.119	^*^	(0.022, 0.216)
PM_z	PRC_z	−0.007	ns	(−0.146, 0.131)
PM_z	AA_z	0.057	ns	(−0.075, 0.190)
PM_z	CC_z	0.131	^*^	(0.024, 0.238)
PM_z	SES_z	−0.065	ns	(−0.154, 0.025)
PM_z	AI_Use_z	0.114	^*^	(0.031, 0.197)
HS_z	PDL_z	0.068	ns	(−0.027, 0.163)
HS_z	AIT_z	0.374	^*^	(0.272, 0.476)
HS_z	PRC_z	0.128	ns	(−0.005, 0.261)
HS_z	AA_z	0.017	ns	(−0.105, 0.139)
HS_z	CC_z	0.116	^*^	(0.023, 0.209)
HS_z	SES_z	−0.050	ns	(−0.130, 0.031)
HS_z	AI_Use_z	0.064	ns	(−0.019, 0.146)
BI_z	PM_z	0.088	ns	(0.000, 0.175)
BI_z	HS_z	0.155	^*^	(0.049, 0.260)
BI_z	AIT_z	0.247	^*^	(0.152, 0.340)
BI_z	SES_z	−0.033	ns	(−0.109, 0.043)
BI_z	AI_Use_z	0.138	^*^	(0.048, 0.227)
WTP_z	BI_z	0.776	^*^	(0.358, 1.196)
WTP_z	HS_z	−0.002	ns	(−0.148, 0.145)
WTP_z	SES_z	0.088	ns	(−0.004, 0.179)
WTP_z	AI_Use_z	−0.038	ns	(−0.157, 0.082)

Beta (Std.) reports standardized coefficients (std.all). Sig indicates whether the 95% confidence interval excludes zero (^*^ = significant; ns = not significant).

As shown in Table 5, all three specified standardized indirect effects were statistically significant based on bootstrap BCa 95% confidence intervals that excluded zero. Specifically, the indirect association SES → PDL → PM → BI (ind_SES_PM_BI) was positive [β = 0.0046, 95% CI (0.0006, 0.0124)], indicating that higher SES was statistically associated with stronger behavioral intention through higher digital literacy and subsequently greater parental mediation. In addition, the indirect association SES → PDL → AI trust → BI (ind_SES_Trust_BI) was significant [β = 0.0140, 95% CI (0.0063, 0.0265)], and the longer chain SES → PDL → AI trust → HS → BI (ind_SES_Trust_HS_BI) was also significant [β = 0.0033, 95% CI (0.0011, 0.0080)], These results are best interpreted as cross-sectional statistical mediation patterns linking SES, digital literacy, trust, relational supports, and behavioral intention, rather than as evidence of temporal or causal mediation.

**Table 5:** Standardized indirect effects (bootstrap, BCa 95% CI).

Indirect effect label	Est. (Std.)	95% CI	Sig
ind_SES_PM_BI	0.0046	(0.0006, 0.0124)	Significant
ind_SES_Trust_BI	0.0140	(0.0063, 0.0265)	Significant
ind_SES_Trust_HS_BI	0.0033	(0.0011, 0.0080)	Significant

Indirect effects were estimated using bootstrap resampling (5,000 draws) with bias-corrected and accelerated confidence intervals (BCa). Estimates are standardized.

### 3.6 Multi-group structural path analysis: socioeconomic differences in structural paths

Table 6 summarizes the multi-group structural invariance test across sample-defined SES subgroups. The regression-constrained structural path model did not demonstrate a statistically significant decrement in fit relative to the configural model (Satorra-scaled LRT *p* = 0.198), and the change in robust CFI was small (ΔCFI = −0.0089; CFI_configural = 0.964 vs. CFI_constrained = 0.955). Together, these results provide limited evidence for SES-based moderation of the overall structural relations, suggesting that the pattern of regression paths is broadly comparable between the sample-defined lower- and higher-SES groups under the tested equality constraints.

**Table 6:** Multi-group structural invariance test across sample-defined SES subgroups.

Model	Chi2 (Scaled)	df	CFI (Robust)	Delta CFI	LRT *p-value*
Configural (Free)	65.89	30	0.964	–	–
Structural (Constrained)	106.47	63	0.955	−0.0089	0.198

Multi-group structural path analysis was estimated with MLR and FIML. The likelihood ratio test (LRT) reports the Satorra (2000) scaled chi-square difference test between the configural and regression-constrained models. Delta CFI is computed as CFI_constrained—CFI_configural.

**Figure 3:**
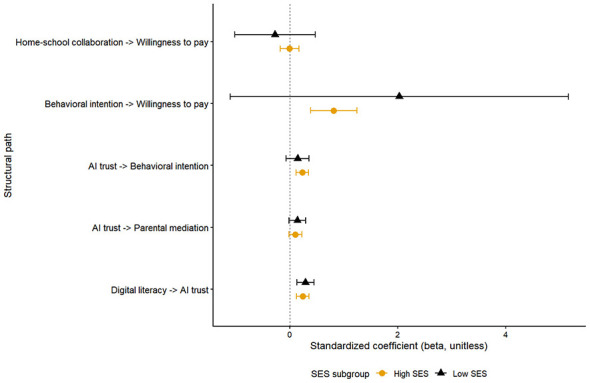
Standardized coefficients for key structural paths by sample-defined SES subgroup. Points represent standardized path coefficients (β) estimated from the configural multi-group structural path model; horizontal error bars indicate 95% confidence intervals. Relative SES subgroups were defined using a median split of the SES index to reflect within-sample variation rather than sharply differentiated socioeconomic strata. The dashed vertical line denotes β = 0. BI, behavioral intention; WTP, willingness to pay; PDL, digital literacy; AIT, AI trust; PM, parental mediation; HS, home–school collaboration; SES, socioeconomic status.

Figure 3 compares standardized coefficients (with 95% confidence intervals) for a focused set of structural paths across the sample-defined lower- and higher-SES groups based on the configural multi-group structural path model. Specifically, the plot contrasts Digital Literacy → Trust, Trust → Mediation, Trust → Intention, Intention → Willingness to Pay, and Home–School Collaboration → Willingness to Pay across groups. The point estimates are presented alongside their uncertainty bounds to facilitate direct visual comparison, and the largely overlapping confidence intervals indicate that between-group differences for these focal paths are generally modest, aligning with the overall invariance evidence reported in the multi-group model comparison.

## 4 Discussion

### 4.1 General discussion: a gatekeeper interpretation of AI adoption in the home learning environment

This study does not support the assumption that the spread of AI tools in households will automatically translate into meaningful educational support. Instead, the structural pattern observed here is broadly consistent with a gatekeeper interpretation in which socioeconomic resources are associated with parents' digital capability and with relational support processes that may help make AI use more governable at home. In this sense, parents are not merely passive adopters responding to a tool's perceived usefulness; rather, they may shape whether AI is incorporated as a scaffold for learning or used more loosely as a convenience. The model explained parental intention more convincingly than willingness to pay, suggesting that financial commitment may be shaped in part by factors outside the psychosocial-relational system captured here, such as household budget constraints, perceived value, pricing structures, school mandates, or institutional expectations. Read cautiously, the present findings suggest that adoption in the AI-enabled home learning environment may be better understood as capacity- and relationship-linked governance rather than as exposure alone.

### 4.2 The usage paradox: frequency does not equate to competence

One of the most consequential findings is that higher household AI use does not consistently translate into broader forms of parental capital or downstream governance capacity, even though it shows a modest positive association with parental digital literacy. This pattern challenges the “digital native” narrative and policy shortcuts that treat access and usage as reliable proxies for competence and governance readiness. In the family context, high-frequency use can just as plausibly reflect passive consumption and convenience-seeking (outsourcing, shortcuts, and automation of routine tasks) as it reflects skill-building engagement. This creates a façade of progress a household looks “AI-active” while remaining structurally under-equipped to verify outputs, set boundaries, or explain limitations to children. The implication is policy-relevant: interventions that prioritize distribution and adoption metrics without investing in capacity-building may unintentionally widen existing gaps, because they expand exposure while leaving the competence gap intact, enabling affluent families to translate AI into educational advantage while less-resourced families drift toward lower-quality, more dependent forms of use.

### 4.3 From competence to relational support: trust as agency, risk as a governance signal

The model highlights trust as a central correlate of parental engagement, but with a critical qualification: trust is not treated here as a soft sentiment or as a demonstrated causal mechanism; rather, it marks an association with parents' reported mediation practices and coordination with schools. This perspective exposes a limitation in strands of the literature that over-invest in risk perception as the principal barrier, implicitly suggesting that amplifying warnings should solve adoption problems. The present evidence suggests a more cautious interpretation: risk-related perceptions may not dominate downstream behavior because they can remain diffuse and difficult to operationalize in day-to-day parenting, leaving families to translate broad concerns (e.g., privacy, output quality, and value alignment) into concrete governance practices with limited institutional scaffolding. A more defensible target is calibrated trust grounded in competence, constraints, and interpretability because fear-based framing can just as easily produce resignation (unchecked use) or prohibition (blanket rejection) as it can produce responsible governance. When parents lack the skills to operationalize caution, awareness may become informational noise rather than a basis for action; when they possess those skills, trust may be one condition under which AI is incorporated with boundaries and accountability.

### 4.4 Mediating mechanisms: capacity-building pathways as the primary route

The mediation results describe how background conditions are statistically linked with behavioral outcomes: socioeconomic resources were associated with parental support chiefly through capacity-building chains in which digital literacy appeared upstream and was linked to intention via trust-centered and relational governance pathways (including mediation and home-school collaboration), with downstream willingness to pay largely appearing through intention rather than direct effects. In contrast, pathways involving abstract concerns about privacy or algorithms appeared comparatively weak in predicting mediation and collaboration once relational processes were included in the model. This does not mean that privacy and algorithmic issues are irrelevant; it means they did not function as the main behavioral correlate in the present specification. Parents may be less stalled by generalized anxiety than by the absence of actionable strategies they can apply in the moment: rules for use, routines for verification, and practices for child-centered explanation. The implication is methodological as well as theoretical: if prevailing measures of awareness primarily capture worry or ambient unease, they may fail to predict governance behaviors; the construct that matters is not merely noticing that systems are opaque, but knowing how to respond to that opacity in everyday parenting.

### 4.5 SES subgroup comparisons: broadly similar structural patterns

The multi-group results suggested broadly similar structural patterns across the sample-defined SES subgroups, but this result should be read as within-sample evidence rather than as a population-level claim about socioeconomic inequality. It should not be taken as evidence that socioeconomic inequality is absent; rather, within the present sample, it indicates limited evidence for strong structural moderation of the estimated path relations. Given the relatively highly educated sample, the non-probability online recruitment strategy, and the median-split grouping strategy, these subgroup findings should be interpreted cautiously and treated as exploratory. It remains possible that socioeconomic inequality operates more through differences in starting levels of resources than through sharply different structural mechanisms, but this interpretation requires direct testing in more socioeconomically heterogeneous samples, especially among lower-SES and digitally disadvantaged families.

### 4.6 Practical implications: moving beyond normative expectations and clarifying accountable responsibilities

A practical implication of these findings is that if parental digital literacy and trust-linked relational governance are important leverage points, then approaches focused only on adoption or generalized risk messaging may be insufficient (Livingstone and Blum-Ross, 2020). Schools should not simply introduce AI and hope parents adapt; they must provide parent-facing infrastructures that translate policy into practice, including task-level guidance (what AI may support vs. what must remain student-generated), routines for verification and attribution, and communication channels that make home–school coordination usable rather than aspirational (Livingstone, 2024; Ni et al., 2025). Developers cannot treat child use as a minor edge case; if trust is to be calibrated rather than coerced, products must offer meaningful parental controls, transparent data practices, and child-appropriate safeguards that reduce the governance burden currently pushed onto households (Shneiderman, 2020). Policymakers, likewise, should resist framing AI-supported learning as an optional premium purchase that families must navigate alone; the combination of modest explanatory power for willingness to pay and the centrality of relational governance signals a need for institutional support that protects children and prevents market design from becoming a new axis of educational stratification (Pitzalis and Porcu, 2024; Wang et al., 2025; Williamson and Eynon, 2020).

### 4.7 Limitations and future directions: toward dynamic, institutionalized family AI governance

Several limitations should be acknowledged. First, the cross-sectional design constrains causal interpretation, and the observed associations should not be read as establishing temporal or causal sequences. Second, several constructs were represented by single-item or brief composite measures, and reliability for the two-item AIT and HS composites was relatively low; these features may introduce measurement error, limit construct validity evidence, and reduce confidence in the stability of some structural estimates. Third, the present analysis used observed composite variables in a structural path model rather than a full latent-variable SEM, which improved parsimony but did not explicitly separate measurement error. Fourth, the SES subgroup comparison was based on a median split within a relatively highly educated, non-probability online sample and should therefore be interpreted as exploratory rather than as evidence of sharply differentiated socioeconomic strata. Finally, willingness to pay was only modestly explained by the model and was measured with a single item, suggesting that economic commitment may also depend on omitted variables such as income constraints, pricing, perceived value, cultural attitudes toward paid educational services, or school requirements. These constraints limit external validity, particularly for families with fewer educational, economic, or digital resources. Future work should therefore use more robust multi-item measures, more socioeconomically heterogeneous samples, and longitudinal or mixed-method designs to examine how family AI governance develops over time (Das, 2023, 2024; Mustafa et al., 2024).

## 5 Conclusion

This study contributes to research on the AI-enabled home learning environment by moving beyond access- and usage-centered accounts toward a gatekeeper perspective centered on parental capacity, trust, and relational support. Drawing on cross-sectional survey data from 567 Chinese parents of children from preschool (pre-K) through secondary school and a theory-informed structural path model, the findings suggest that socioeconomic advantage is associated with supportive engagement with educational AI primarily through parental digital literacy, which in turn is associated with stronger trust and relational support processes, including parental mediation and home–school collaboration. Frequent household AI use did not consistently relate to broader digital capital or governance readiness, even though it showed a modest positive association with parental digital literacy. The results further suggest that background conditions are linked to downstream support mainly through capacity-building and relational pathways, whereas risk-related constructs appeared less central in predicting mediation and collaboration within the present model. Although the model explained more variance in behavioral intention than in willingness to pay, and financial commitment likely depends on additional market and institutional constraints, the subgroup analyses suggested broadly comparable structural patterns across the sample-defined SES groups. Taken together, these findings indicate that equity-oriented AI-in-education initiatives are unlikely to succeed through device distribution or adoption metrics alone; they may also require efforts to strengthen parents' operational competence, calibrated trust, and institutional support for responsible family governance of AI in children's learning.

## Data Availability

The raw data supporting the conclusions of this article will be made available by the authors, without undue reservation.
